# How Happy Are Equine Athletes? Stakeholder Perceptions of Equine Welfare Issues Associated with Equestrian Sport

**DOI:** 10.3390/ani11113228

**Published:** 2021-11-12

**Authors:** Tamzin Furtado, Liane Preshaw, Jo Hockenhull, Jennifer Wathan, Janet Douglas, Sue Horseman, Rebecca Smith, Danica Pollard, Gina Pinchbeck, Jan Rogers, Carol Hall

**Affiliations:** 1 Leahurst Campus, University of Liverpool, Neston, Liverpool CH64 7TE, UK; tfurtado@liverpool.ac.uk (T.F.); rebecca.Smith@liverpool.ac.uk (R.S.); ginap@liverpool.ac.uk (G.P.); 2 The Horse Trust, Slad Lane, Princes Risborough, Bucks HP27 0PP, UK; liane@horsetrust.org.uk (L.P.); jan@horsetrust.org.uk (J.R.); 3 Animal Welfare and Behaviour Group, Bristol Veterinary School, University of Bristol, Bristol BS40 5DU, UK; Jo.Hockenhull@bristol.ac.uk (J.H.); sue.horseman@bristol.ac.uk (S.H.); 4 The Brooke Hospital for Animals, London EC3A 2BJ, UK; jennifer.wathan@thebrooke.org; 5 World Horse Welfare, Anne Colvin House, Snetterton, Norwich NR16 2LR, UK; janetdouglas@worldhorsewelfare.org; 6 The British Horse Society, Abbey Park, Stareton, Kenilworth, Warwickshire CV8 2XZ, UK; dee.pollard@bhs.org.uk; 7 National Equine Welfare Council, Slad Lane, Princes Risborough, Bucks HP27 0PP, UK; 8 School of Animal, Rural and Environmental Sciences, Nottingham Trent University, Nottingham NG25 0QF, UK

**Keywords:** horse, equine, welfare, equestrian sport, competition, riding, training, quality of life, horse-human-relationship, qualitative research

## Abstract

**Simple Summary:**

The welfare of horses within equestrian sport is increasingly being scrutinised by both the public and those involved in the sector. To identify the main concerns and discuss the potential to improve the welfare of these equine athletes, a workshop involving participants from equestrian sports and animal welfare research was held. Participants concluded that the main challenges in equine welfare arise from conflicts between competition demands and the basic needs of the horse. To enable those involved in equestrian sport to monitor the impacts of management, training, and competition on the welfare of equine athletes, the use of formal welfare assessment tools was discussed, alongside interventions which would promote positive welfare across equine athletes’ lives.

**Abstract:**

The international governing body for equestrian sports, the Fédération Equestre Internationale (FEI), states that the welfare of the horse must be paramount and never subordinated to competitive or commercial influences. However, there is growing unease about welfare issues from both within and outside the sport. The aim of this study was to understand stakeholder perceptions of current welfare issues within equestrian sport, determine whether there is scope for change, and explore attitudes towards welfare assessment. Participants (*n* = 48) from equestrian sport (*n* = 38) and animal welfare research (*n* = 10) attended a workshop that included welfare-related presentations and focus group sessions. The focus group sessions were recorded, anonymised and analysed using thematic analysis. Conflict between the demands of competition and the needs of the horse was identified as a key welfare challenge. Although the physical health of equine athletes is closely monitored, horses’ psychological needs are sometimes overlooked. Participants recognised that improving competition practices may not be as impactful as improving the general management and training of horses. The term “quality of life” was considered preferable to “welfare”, which had negative connotations. Participants appreciated the idea of incorporating formal welfare assessments into their training and competition plans but stated that existing tools are rarely used and are not deemed feasible for real-life conditions.

## 1. Introduction

The Fédération Equestre Internationale (FEI) is the world governing body for the equestrian sporting disciplines of Jumping, Dressage and Para Dressage, Eventing, Driving and Para Driving, Endurance, Vaulting and Reining [1]. The FEI Code of Conduct states that the welfare of the horse must be paramount and must never be subordinated to competitive or commercial influences [2]. The FEI does not define what it means by the term “welfare”, but for the purpose of this paper, the authors use the definition by Webster [3] that welfare is the animal’s perception of its own physical and emotional state [3]. There is growing unease about compromised welfare in equine athletes, not only from the public and media, but also from within the equine sector [4]. 

Scientific thinking about what welfare represents has advanced significantly over recent years, with biological functioning, a natural lifestyle, interactions with humans and the animal’s resultant mental state all being deemed important [5,6,7]. There has also been a move towards providing animals with positive experiences and identifying signs of good welfare, as opposed to just avoiding negative welfare states [5,6,8,9,10,11]. However, behavioural signs on which to base such evaluations are inconclusive [12]. Moreover, the term “animal welfare” can mean different things to different people [13], and this affects how it is assessed and the conclusions drawn [14,15]. Such differences, as well as other barriers to change, must be considered in initiatives aimed at improving the lives of animals, including equine athletes [16]. 

At the 2004 Global Dressage Forum, there was discussion of the amendment to the FEI dressage rule 401.1, that the aim of dressage training should be to produce a happy (equine) athlete [17]. This amendment has been retained within the 2021 edition of the FEI Dressage Rules [18], but how the happy equine athlete is conceptualised by the FEI is not clearly stated. Reference is made to desirable performance attributes, such as “displaying a natural and harmonious balance both physically and mentally” [18], but there is no clear definition or improved guidance to support the understanding of what constitutes a happy equine athlete, so evaluation can only be based on subjective judgement. Interpreting the behavioural signs of underlying mental state in ridden horses is challenging, and the conclusions drawn have been found to vary according to the role and experience of the observer [19]. Further behavioural evidence of underlying equine mental state is required, particularly signs of a positive mental state [12]. 

Various scientific tools have been developed to support valid and reliable welfare assessments, including the Animal Welfare Indicators Network (AWIN) welfare assessment protocol for horses [20]. However, existing welfare assessment tools are not widely used [14], and stakeholders may have a limited understanding of the purpose of equine welfare assessment within the industry. To help stakeholders conduct valid and reliable welfare assessments on their horses throughout their lives, the use of available tools by owners, riders, trainers etc. needs to be feasible under everyday conditions. To achieve this, it is important that researchers understand other stakeholders’ perceptions of the currently available welfare assessment tools and whether they would be prepared and/or able to use them in practice. 

Concerns surrounding the welfare of equine athletes may result in ethical dilemmas for stakeholders within equestrian sport, as well as calling into question the ethics of using horses in sport at all [21]. Prioritisation of equine welfare is essential to maintain our ethical obligations to our equine sporting partners. Only by dynamically assessing the welfare of horses during sports participation and beyond can equestrian sport remain within society’s moral and ethical frameworks of acceptable animal use [22]. Furthermore, it is important to ensure the sustainability of equestrian sport which is dependent upon societal acceptance of horses as athletic partners in competitive scenarios [23]. The Social Licence to Operate (SLO) (an intangible, unwritten social contract that is not legally binding) [24], is “granted” when an activity is considered sufficiently legitimate and trusted by the wider community, and when it operates with the implicit consent of that community [25]. This concept has recently been discussed in relation to public concerns surrounding the welfare of horses in equestrian sport [23,24], and these concerns must be addressed to both improve the welfare of equestrian athletes and to secure the future of the sport. Sport-related risks to horse welfare and equestrianism’s SLO have also been brought into sharper focus by recent changes in communication technology. For example, social media is more likely to act as a forum for raising concerns over sports horse welfare than conventional print-based media releases [26].

Within equestrian sport there are undoubtedly discipline-specific differences in both the welfare of the equine athletes involved and in the public perception of each type of equestrian sport. In the UK, the strategic plan for the welfare of horses bred for racing acknowledges the need for change and for taking responsibility for them before, during and after their competitive careers [27]. No definitions of what is meant by “quality of life” or “welfare” are provided within the racing strategy document, but it is stated that the former fits closely with standard definitions of welfare [27], which is in line with how many researchers view these concepts [28,29]. The future sustainability of each equestrian discipline depends on how it responds to challenges to its SLO [23] and whether it can demonstrate how the lifelong welfare of its equine athletes is being prioritised.

The aim of this study was to gain a better understanding of stakeholders’

- Perceptions of current welfare issues within selected equestrian sports (dressage, showjumping, eventing and endurance);
- Attitudes towards assessing sport horse welfare and the currently available welfare assessment tools;
- Views on how change can be brought about to improve common areas of concern around equine welfare.

## 2. Materials and Methods

Qualitative methods were deemed most appropriate to explore this under-researched area, as they allow researchers to gain insight into participants’ experiences through open questions [30,31,32]. Focus groups were used to gather data, as they enable researchers to explore participants’ views on an issue via their discussions, interactions and experiences [33], whilst also enabling participants to consider and reflect on information shared by others.

This project was approved by the University of Liverpool’s Veterinary Ethics Committee (reference VREC1057). This committee reviews all research submissions (including sociological research) within the University of Liverpool Veterinary School.

The focus groups were run within a wider virtual workshop about sport horse welfare entitled “How Happy are Equine Athletes? Assessing Equine Quality of Life in Equestrian Sporting Disciplines”, which took place on 30 March 2021 using a combination of the Zoom webinar platform and Zoom meetings (to facilitate the focus groups). The aim of the workshop was to provide a platform for discussion and collaboration between representatives of equestrian sporting disciplines and those involved in the assessment of animal welfare and quality of life. 

The workshop objectives were to

- Gain a better understanding of the current practices and guidance within equestrian sporting disciplines that protect or improve equine welfare;
- Evaluate the potential usefulness of current approaches to assess animal welfare, and inform the development of measures relevant to the equestrian sports sector;
- Plan future collaborative initiatives to promote the consideration of equine welfare in the sporting context and provide evidence of how to recognise a “happy equine athlete”.

All participants were invited to watch a series of presentations about different aspects of sport horse welfare, ranging from social licence to operate and the ethics of equine sport, through to considerations of behavioural signs of affective state, patterns of equine sleep and measures/tools used to assess welfare. There were also presentations from representatives of each of the four main equestrian sports considered at the workshops (dressage, eventing, showjumping and endurance), all of whom used video footage to describe their horses’ competitive lives and to talk about their experiences and perceptions of equine welfare within their discipline. This format meant that all focus group participants were able to watch a range of talks and case studies of different aspects of equestrian sport, promoting subsequent rich discussion. See the supplementary information (Table S1: Workshop programme) for the workshop programme and format.

The workshop was funded by and advertised via the Animal Welfare Research Network (AWRN) and promoted by the National Equine Welfare Council (NEWC) and contacts across equestrian sport (including the governing bodies for each sport who were notified and invited to share the workshop invitation). Focus group participants were then selected from workshop attendees on a “first-come, first-served” basis and their involvement in equestrian sports or animal welfare research. The total of six focus groups included four equestrian discipline-specific groups (endurance, showjumping, dressage and eventing), one mixed equestrian sports group (for other equestrian disciplines including racing, western riding and polo), and one group consisting of welfare/research staff. See Table 1 for details about the focus group participants (please note that some details have been slightly altered to protect anonymity, given the relatively small pool of participants competing at this level in each sport). All participants were supplied with an information sheet prior to confirming their participation and a form to sign to confirm their consent to the recording of the focus group sessions and subsequent use of the data. At the start of the session, participants were briefed on the use of the technology involved and asked to respect each other’s views and anonymity. 

The focus groups for all disciplines were run concurrently as part of the workshop schedule. Each focus group was split into two one-hour sessions (i.e., twelve hours in total), and each was run by an experienced facilitator (TF, JW, DP, JH, RS and SH). The facilitators worked collaboratively to develop and pilot the focus group content. The format for each group discussion was the same, regardless of the group’s discipline, although some questions differed slightly between groups. To ensure maximum opportunity for participation despite the online format, each group used a “Jamboard”. This is an online, open-access whiteboard that participants can use to add their thoughts and ideas around the discussion topic, as well as speaking out loud. The focus group plan is provided in the supplementary information (Table S2: Focus group plans). 

The focus groups were recorded using Zoom’s audio-record facility, and screenshots were taken to capture the Jamboards. Immediately following each session, all facilitators shared notes about the main themes and ideas that had arisen in each group. They also shared their experiences in a “wash-up” meeting. These additional data helped to ensure that the audio recordings and screenshots had fully captured the feel and ethos of the discussions in each group. 

**Figure 1:**
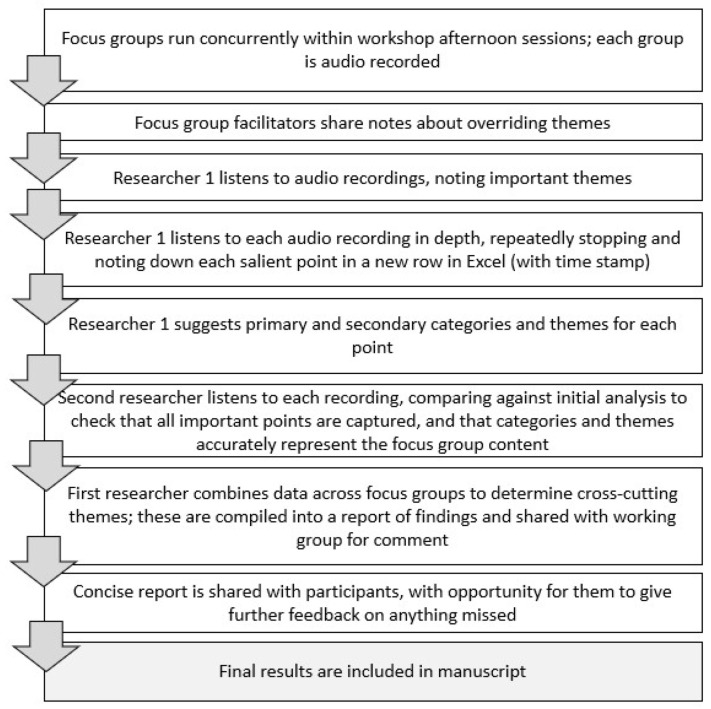
Step-by-step stages followed in the analysis of the focus group data.

To analyse the data, “live coding” was used (i.e., rather than directly transcribing data, recordings were simultaneously listened to, noted and coded). See Figure 1 for the step-by-step stages followed in the analysis of the focus group data.

This method is considered particularly useful for focus group analysis, because it allows analysts to become intimately acquainted with the recording and participants’ tone of voice, verbal cues, etc., aspects that can sometimes be missed during the process of transcription and creation of a “dry” transcript [33,34]. In this process, one researcher (TF) first listened to each recording, writing down ideas or parts of the discussion that were particularly pertinent, and checking those against the Jamboard data and facilitator notes. Subsequently, this researcher listened closely to each recording, stopping the recording and summarising each point made by participants; notes were transcribed verbatim when the specific language used was particularly interesting or insightful. Each item was listed per-row in Microsoft Excel (2010) and time-coded to enable easy return to each quote. Following this stage, the same researcher listened to the recording again to ensure that all salient points had been captured, this time adding ideas for categories and themes for each item in another column in Excel. Categories were developed iteratively according to the ideas discussed by participants and were often revised according to new or later data. Each group’s facilitator then listened to the recordings, checking that all the important points had been captured during this process and suggesting changes where necessary to the categories and themes. 

The data from each individual group were then combined to determine which ideas and themes cut across all groups and which were unique. Cross-cutting themes were brought together into a short report, which was sent to the participants to enable them to comment on areas that they thought had been misrepresented or could be clarified. No additional changes were made after this point.

## 3. Results

While each group discussed some discipline-specific issues, the data showed that participants considered the underlying issues affecting sport horse welfare to be broadly similar across equestrian sports. In this paper, we focus on those universal themes, while noting discipline-specific challenges that were identified within individual groups. Generic welfare challenges predominantly centered on the conflict between the innate needs of horses and the demands of competition. The focus group data clearly described how the evolving perception of equine needs is leading to a change in the way in which some stakeholders view equestrian sport, and that this may impact societal acceptance and social licence. This conflict was described as most apparent when the horse was part of a commercial equine business, as is often the case for competition horses who may be bred, trained, competed and cared for as commodities by a complex network of stakeholders. In these results, we detail the main welfare issues that were perceived to arise in sport horses because of their participation in equestrian sports, participants’ attitudes to assessing sport horse welfare and their views on the currently available welfare assessment tools, and how change can be brought about to improve common areas of concern in relation to the welfare of equine athletes.

### 3.1. Responsibility for Equine Athletes

Participants across the focus groups described an inherent sense of responsibility around involving non-human animals in human sports. Equestrian sports are traditionally built on the assumption that horses are consensual partners in sporting disciplines, but participants in the focus groups recognised that there may be conflict between human and equine choices regarding participation.

Eventing: *“We have this element that we are pushing another animal to step up to the mark and make that win happen. There are moral grounds of do we really Go On! or don’t we? I don’t know.”*

Welfare/researchers: *“It was really heartening in the morning talks… asking should we be asking if horses wanted to take part in these activities. I think it is important we take the horse’s preferences into account, but there’s a risk the horses would say no. In terms of social licence, maybe that’s an important discussion to have.”*

Discussions throughout all groups were based around lessening ethical tensions and equine welfare compromises that may arise for the varied stakeholders involved in equestrian sport to ensure that equine participants experience good welfare throughout their “career” in sport and in retirement.

### 3.2. Life as a “Horse” versus Life as an “Equine Athlete”

Across the disciplines, participants described how there may be tension between the needs of the horse “as a horse” (i.e., its ethological needs) versus the demands of life as an equine athlete. Horses were constructed as having innate needs such as social contact, freedom (e.g., regular paddock turnout) and choice of activity. Participants agreed that, ideally, the needs of a horse and the needs of a sport horse should be considered to be the same.

Mixed sports: “*They might be trained for one hour a day, but what’s happening the rest of the 23 [hours] a day. What are they doing—making sure it’s species appropriate? A companion pony might have different requirements to a dressage horse, but the 23 h of a day they’re a horse.*”

However, the demands of life as a competition horse are such that providing for a horse’s ethological needs (for example, social contact, turnout and choice were discussed) requires additional input (and associated workload) from the human caregivers and is perceived to present risks to the horses themselves (for example, field injuries). This is problematic for animals whose value relies on them being in a state of optimum health. Life as an equine athlete also includes other demands that are unlikely to be viewed as positive experiences by the horse, such as extensive land and air travel. As a result, participants stated that equine needs are sometimes compromised to allow the horse to participate in equestrian sport, and they sometimes feel uneasy about the consequences of these compromises.

Welfare/research group: “*A lot is expected of them—the demands of the sport, being away from their social contacts, crossing the Atlantic. I guess they must be getting an adrenaline rush from some sorts of sports. How do we know they really enjoy it? If we gave them choice, would they choose to do that competitive sport, or just prance around the field and play with their friends?*”

Participants also discussed the fact that, for most equine athletes, the focus of equine care is on their physical health, rather than their mental or emotional health, and this was considered problematic.

Mixed group: “*I think it’s important that the emotional, mental side is given as much importance as the physical. Physically, they’re peak athletes, but possibly the mental side is not given the same support. You could argue the same with human sports as well. There’s obviously a conflict there.*”

The view that the psychological health of sport horses is an area of significant welfare concern was discussed across groups. 

An interesting difference in the perception of welfare was discussed in relation to the level of competition. Each group spontaneously brought up the issues with different levels of care and training/riding skill provided at different levels of equestrian sport. Participants perceived that at lower-level competitions, horses are treated more like “horses” than like athletes. They might have better welfare in some ways (e.g., needs such as social contact and turnout are more likely to be met), but other aspects of their welfare might be compromised due to having non-professional ownership and care (e.g., poorly fitting tack). Horses competing at a higher level were perceived to have poorer welfare in terms of psychological health (e.g., social isolation) but better welfare in relation to physical health, nutrition and horsemanship.

Showjumping: “*I love watching showjumping, but if I go to a local BS [British Showjumping] show, I sit there watching this [hands over eyes]. Being educated does make you see stuff you wish you didn’t see anymore, and you could just enjoy the sport*” [laughs]

Endurance: “*Leisure horses are often overweight, their saddles aren’t checked, there’s no balanced diet, they don’t need to see the physio because they’re “just a leisure horse” and deemed not to need extras.*”

### 3.3. The Demands of the Equestrian Sports Industry

Participants discussed the difference between horses produced privately versus horses who are effectively part of a complex production system in which the successful equine athlete and competition results are the products. Sports with higher amounts of investment and prize money (e.g., racing, dressage and showjumping) were generally considered more problematic in this regard than others (e.g., eventing or endurance), although this was not always the case.

When horses are produced as part of a professional system, participants felt that there is sometimes a need to push horses harder, or more quickly, than might otherwise be ideal, for example, to prepare them for young horse classes, or when moving up to higher levels of competing.

Showjumping: “*Young age classes are a showcase for breeders, but it’s counter intuitive in terms of longevity.*”

Eventing: “*As horses move up the levels, we inevitably are going to be pushing them physically and mentally and it’s going to sometimes be a struggle.*”

Participants felt that it was generally inappropriate to compete horses at young ages, but this was perceived to be part of the culture of competitive riding due to the demands of the competitive stakeholder network. 

Furthermore, the production of young horses was perceived as generating excess horses that are no longer part of the competitive system for one reason or another (for example, horses that become injured during training, cannot cope mentally with training, or are retired). However, it is not clear who, if anyone, is responsible for the welfare of those horses outside of their competitive careers. 

The industry supporting equestrian sports means that there is a complex network of stakeholders surrounding each individual horse, each yard and each sport. Those stakeholders include riders, trainers, owners, farriers, vets, grooms, judges, competition stewards, physiotherapists, the media and sporting bodies. Each stakeholder can place conflicting demands on other stakeholders within the network, which can adversely affect a horse’s welfare.

Mixed group (racing participant): “*I’ve often tried to describe the job of a trainer as a mix of being a football manager, a headmaster at a private school and a cattle farmer, dairy farmer. In that you’re looking after someone’s very expensive [child/horse]—that’s your headmaster—and you’re judged by their sporting activities. And we’re very lucky in racing we get so much media coverage, but with media coverage you get so much pressure. And thirdly, you’re a dairy farmer because you are in charge of a production animal that has to produce—it’s trying to balance those three without compromising the horse’s welfare. But inevitably there will always be compromises somewhere.*”

Showjumping: “*There’s also a vet–owner–trainer decision matrix, and with commercialisation, decision making isn’t based on one person, it’s a team. Within that team there’ll be competing demands…where does the horse sit in that?*”

As described by these participants, the complex network of demands around competition horses was perceived to result in pressure on the horses themselves, leading to, for example, horses being pushed into competition before they are ready. Participants described themselves as needing to speak up and be the “voice” for the horse, stating that it is their job to protect horses from the demands of competition by making individual decisions about what they think is, or is not, acceptable for each horse.

Dressage: “*We all choose our own line, how far we’re prepared to push. It’s incredibly important we know when to stop.*”

The discussions also clarified that there is little support for people who make decisions in the horses’ best interest in cases where a decision is considered unusual or does not conform to the equestrian norm. This was not just the case for riders but also for other stakeholders, for example, the dressage group spoke about the need for stewards and judges to be comfortable with being “unpopular” if they were to speak up against well-known riders. There was also clear understanding across all groups that welfare at competition is only one very small component of sport horses’ lives. This is problematic because yards are perceived as places that are entirely unregulated and mostly untouched by the rules of formal competition. For example, specific tack or ways of riding might be disallowed during competition, but horses can be cared for and prepared for competition in any way that their owner, rider or trainer sees fit.

Eventing: “*Competitions are a showcase—they’re what you see in the window, what the public sees. But what is happening before that at home and in training? That is more important.*”

### 3.4. Assessing the Welfare of Equine Athletes

Across the groups, there was much discussion over whether we could assume that horses experience “happiness” in the same way that humans might experience it, and it was generally considered to be a problematic and unmeasurable concept, particularly because, as one participant suggested,

Eventing: “*none of us, and none of our horses are happy all the time or having fun all the time—it’s not just about having fun. Are our horses experiencing a life worth living over a timespan?*”

Some participants also considered it difficult to think about “happiness” specifically in relation to their horse’s competitive life.

Endurance: “*I don’t think I’ve ever thought when I’ve been going along in a race “is my horse happy?” I go along thinking about looking after them, is he eating and drinking, is he comfortable, is he normal? But I haven’t specifically thought is he happy, is he sad, is he miserable?*”

Similarly, the idea of assessing “welfare” was perceived as problematic because of the inherently negative connotations of the word itself.

Showjumping: “*When you think of welfare, it’s bad, you must stop bad welfare, but maybe it’s just the reading I’ve been doing, but in the last few years, there seems to be a shift towards positive welfare…Maybe that’s a problem with why people have a stigma about talking about welfare, that traditionally it’s associated with being bad.*”

Participants were open to the idea of assessing constructs that are scientifically synonymous with welfare (i.e., constructs that involve assessing indicators of physical and psychological health), but the term welfare was typically associated with negative connotations. It was felt that an alternative term, such as quality of life, would be more likely to encourage engagement. Most groups discussed the need for more research before welfare can be formally assessed by stakeholders, including how this would be measured, how change over time would be accounted for, who would do the measuring, how could consistency be ensured and which aspects would be measured? Participants in the welfare/research group described the difficulty of applying welfare assessment tools developed for the general horse population to competition horses. Existing welfare assessment tools predominantly include measures of physical health, but as competition horses are often in optimum physical health, compromises to their psychological health may not be adequately captured.

The discussions also highlighted a paradox in assessing welfare, in that there is a need to be familiar enough with the horse to identify any changes but that the assessor also needs to be objective enough to view the horse as an unbiased observer. Participants frequently described individual behaviours or situations that, because of their familiarity with the individual horse, led them to believe they knew whether the horse was well or “happy”. Participants also recognised that knowing the horse well meant that it was harder for people to be objective about their decisions.

Endurance: “*The only way formal assessments would work is if you had an independent person to assess your horses, because it’s really hard to step back and look at your set up. I’m sure we all believe we’re doing the best for our horses, and even if we’ve made a compromise here, it’ll be because of a financial resource, or land or whatever—we all think we’re doing the best we can with what we’ve got, so to then be able to objectively say, let’s look at the parameters and measure how happy our horses are would be really hard. The only way would be to have someone entirely independent who came and observed your horses in their environment for quite a long period of time.*”

Participants also acknowledged the need to be context-specific when conducting behavioural assessments., for example, recognising that the same behaviour might signify different things during competition compared to at home.

Eventing: “*The idea of QBA [Qualitative Behavioural Assessment] and quality of life measures needs to be context specific—what some people see as excitement, if you look from a behavioural indicator perspective, you might say it’s a classic stressed horse appearance. But, actually, in context, is it the horse ready and raring to go? I know I’m going to gallop cross country. This whole seminar, everything needs to be considered within the context of the individual sport, that’s really important. We’ve got to be careful using behavioural assessments, particularly when talking to the general public who don’t understand the nuances.*”

Participants, therefore, appreciated the idea of incorporating welfare assessment into their training and competition plans but found it hard to imagine how this could be feasibly achieved:

Eventing: “*I don’t know if it’s something that’s feasible to incorporate, I love the idea of it, but I question the realistic application of it.*”

Few participants formally measured any aspects of their horse’s welfare, though all groups discussed that they continually monitored their horses on an informal basis and would know when there was something wrong.

Endurance: “*You have a tolerance level of what you perceive to be normal based on the experiences of all the qualifications and races you’ve done in the past. When you get a new horse or young horse. that’s when you get the most variation.*”

One endurance rider discussed keeping a training log to track her horse’s progress and other factors. including weather and tack changes. One eventing participant had completed a formal welfare assessment for her own horses, and one racing trainer described keeping a log for exercise riders to note how each horse felt each day. However, aside from these examples, the competitive focus group participants did not formally monitor their horse’s welfare. 

Because of the paradox of assessors needing to be both objective and know the horse intimately, the complex network of stakeholders surrounding each individual horse could also be beneficial for safeguarding welfare, because of the range of views that each stakeholder might have. Endurance riders discussed the idea that the endurance crew (people who meet the horse at regular check points and assist the rider during vetting) is well-placed to provide this support to endurance horses, as they are very closely engaged in assessing the horse’s health during competition and can be more objective than a rider might be during a competition. 

In terms of assessing welfare at events, both dressage and endurance already incorporate feedback from objective and trained observers (judges during dressage and vets in endurance). However, even though dressage judges receive specific, rigorous training and should deduct marks for conflict behaviours, such as tail swishing, the dressage group did not feel that having a judge as an objective observer means that their horses are protected from poor welfare or poor riding.

Dressage: “*At Pony Premier Leagues, I remember a kid who did really well, her reins were so tight, and her pony was so tight, and she won. I remember thinking, if that’s the way you have to ride to do well at pony FEI, I don’t want to do it. I couldn’t understand why she’d been given those marks. If there was more reasoning, that would help riders as well.*”

The dressage group also discussed the need for stewards to observe the warm-up arenas to ensure that horses were ridden appropriately outside of the competition arena, particularly in relation to excessive flexing of the neck and poll (termed hyperflexion). Both the dressage and showjumping groups mentioned the need for stewards to have training and support in knowing when and how to intervene in difficult situations.

In contrast, endurance competition involves multiple veterinary inspections (pre-ride, at 30 km intervals, and post-ride). These include the measurement of heart rate, a lameness/muscle soreness check, and metabolic fitness to continue. This was perceived as helping endurance riders to familiarise themselves with their horses’ physical health more closely than in some other sports, though there was recognition that the tested parameters are not necessarily able to detect psychological stress.

Endurance: “*I think, as endurance riders, we are quite good at picking up the physical signs–high heart rate, recovery times. But I think it’s more the mental side of it. My Arab can have a very low heart rate, but he can be still stressed, so I think it’s learning the behavioural signs a bit more as well.*”

Nevertheless, the endurance group felt that there could be welfare issues, both at competition and at home, that are not captured by those examinations.

The welfare/researchers group discussed their experience with assessing welfare measures in other settings, such as in small animal veterinary medicine. This group discussed, at length, the difficulty in encouraging the uptake of such measures, particularly for routine use rather than to monitor a specific disease or during end-of-life care.

Welfare/research group: “*Pain scores have higher uptake. Subjectiveness of the term ‘quality of life’ may be one of the issues with getting people to do it People think ‘I know my dog is happy, I don’t need to fill in this form’ or as a vet ‘I have experience, I have a degree, I don’t need this form’. I think with pain scores, they have a higher uptake. Possibly people think pain is more measurable.*”

### 3.5. Public Social Licence in Equestrian Sport

Participation in equestrian sports means that the horses involved are visible to various audiences, ranging from other sporting participants through to the general public. All groups discussed the pressure of being visible to the public and considered that the concept of “social licence to operate” provides an important opportunity to ensure that the ethics and practices of equestrian sport evolve. The racing industry provided a useful focus for this discussion, given that racing is under considerable public scrutiny, partly because of its high level of visibility. However, the equestrian sports included in the focus groups were less publicly visible (for example, not routinely shown on television):

Showjumping: “*Showjumping isn’t something non-horsey people watch, it’s not on TV, in newspapers, bar the Olympics—goes under the radar. Therefore, it’s up to us to do the job of the public in terms of social licence to operate, so we become a stronger voice within the industry, pick up the things that are suboptimal for welfare in the same way that Joe Public is doing for racing.*”

Participants felt that social licence is nuanced differently depending on the audience. Many equestrian sports are subject to pressure from within the equestrian community rather than from the general public. It was felt that, while the public has views on what is or is not acceptable, those views are not always representative of the things that are “real” issues in each sport:

Dressage: “*The inside issues are more technical than the things the public grasp onto, like nosebands, things that are very visual. But the subtle things would solve some of the more visual issues.*”

Mixed group: “*The things mentioned in other disciplines—whips, spurs, tight nosebands—these were all things seen in the public eye that come to the attention of the public as signs of negative welfare and while rightly so, it almost takes the spotlight off the other 23 h of the horse’s lives and how they are being managed the rest of the time when they are not in the public eye.*”

This last quote summarises one issue mentioned by the groups: that social license often focusses on visible issues at competitions, and that these reflect only a tiny portion of each horse’s life. Social licence to operate was therefore considered an important factor for encouraging change in the industries surrounding equestrian sport, but overall, participants felt that it was up to the competitive communities themselves to determine which issues have the greatest negative impacts on sport horse welfare.

### 3.6. The Case for “Cautious Optimism”

While the groups discussed many welfare issues in equestrian sport, as outlined above, they also noted that considerable progress has already been made. Examples cited by participants included tightening and increased enforcement of the rules surrounding use of the whip in racing in the UK; the FEI ban on hyperflexion in dressage; a reduction in the prevalence of inappropriate feeding practices in leisure horses; increased awareness of the importance of turnout and socialisation for horses; the inclusion of healthiest condition awards at endurance events; and an increased level of knowledge in the general horse owning population. Accordingly, one participant described that there are grounds for optimism about the future for equine athletes:

Mixed group: “*My own experience, despite the worries and concerns, there is cause for cautious optimism…The attitudes of owners now compared to attitudes 20 years ago, there’s been a marked improvement. There needed to be, but I do think a lot of work has been done, so there’s cause for some optimism.*”

In terms of moving forward with improvements to equine welfare in competition, participants discussed a range of ideas, including showcasing positive welfare; providing better education for grassroots competitors and judges/stewards; giving support to stakeholders who “speak out” about welfare; and making use of social license as a catalyst for bringing about positive change. 

## 4. Discussion

In the focus group discussions, stakeholders within equestrian sports described the need for improved welfare for equine athletes in all disciplines. All participants felt that the majority of those involved in equestrian sports were doing their best to prioritise the welfare of their horses, but that compromises were often made because of various constraints. This conflict was often exacerbated by the varied opinions and requirements of the different stakeholders involved in the production of equine athletes. At the elite level, it is common for a group of stakeholders to be involved in the ownership, training and competitive career of a horse. As noted by McGreevy and Murphy [35], financial investment may lead to the horse being viewed as a commodity, and welfare-related compromises may be made for the sake of gaining returns on investment. In racing, show jumping and dressage, where horses are valued highly in monetary terms, there is more financial investment, and potentially higher financial incentives for performance success, it was suggested that demands placed on the equine athlete are greater, which could lead to further welfare compromises. As well as acknowledging the potential threat of adverse public opinion on the sustainability of sports involving animals [23,24], participants spoke of their need to be the ‘voice for the horse’ and their own sense of responsibility towards protecting the welfare of equine athletes. 

Public debates over specific sport-related issues, such as the use of over-tight nosebands in dressage [36], may not be viewed as key issues by those within the sport but are likely to act as catalysts for improved equine welfare therein. There was recognition of public and media discomfort relating to the use of animals in sport, as noted by Campbell [4], with those sports attracting the most media coverage being most susceptible to criticism. Negative views on equestrian sports are more likely to be voiced online and on social media, particularly by those outside of the equestrian sporting community, rather than in traditional print media [26]. The extensive coverage of racing by the media was viewed as both an advantage and a threat in terms of public support for the sport. In recent years, racing, more than any other equestrian sport, has been under the spotlight in terms of compromised equine welfare and threats to its SLO [23,24,37]. The comparative absence of media coverage of sports such as show jumping was thought by participants to explain why these had attracted less criticism than racing. One positive outcome for racing has been that the need to be seen by the public to be addressing concerns has led to the publication of the racing welfare strategy [27]. To protect the future of other equestrian sports, a similar approach is warranted. 

The FEI rule books clearly state that the welfare of the horse must be paramount and that horse welfare must never be subordinated to competitive or commercial influences [2]. However, there is currently no means of assessing or enforcing this. Conflict between those trying to protect the welfare of a horse and other stakeholders was identified as a major challenge, potentially posing an ethical dilemma for specific stakeholders, including riders and veterinarians [21]. This is important from a social license perspective as the public may not appreciate that the rider is not solely in charge of making decisions about the horse’s welfare and that the decision-making process is much more complex. The focus group participants identified the rider as generally being best placed to regulate the workload and challenges set for the horse, but that support from other participants who were involved with the horse in a non-ridden capacity (for example, grooms and crew) should also be considered. 

The opinions of other stakeholders were seen by participants as being likely to influence riders’ decisions to compete young horses before they are mentally and/or physically ready. The focus group participants were, in general, critical of young horse classes, stating that they could result in an overproduction of horses, cause horses to be pushed harder than they would otherwise be, and reduce the longevity of their careers. These views contrast with the results of studies that retrospectively analysed training and competition records for race and sports horses, which suggested that those trained and competed from an earlier age have longer and more successful careers [38,39,40,41,42,43]. However, because these studies were retrospective, they cannot provide information regarding the nature and type of exercise provided and their associations with career longevity. Furthermore, these studies did not investigate the effects of early training and competition on the physical and mental stress experienced by young horses. Insufficient preparation has been linked to the development of behavioural signs of mental and/or physical stress, including some conflict behaviours, in both dressage and show jumping [44]. Further research is needed to determine the optimal age for horses to commence different types of training/competition, and the form that these take, but more gradual and comprehensive preparation for the future physical and mental demands of equestrian sport would be advantageous [45]. 

There is no current requirement for data relating to competitive longevity and, importantly, the post-competitive care of ex-equine athletes (including those horses that failed to make the grade), to be collated. Although focus group participants agreed that safeguarding the welfare of equine athletes throughout their lives is an important consideration, it is unclear whose responsibility this is or how they should be protected. Addressing responsibility for the long-term care of equine athletes should be a priority for all equestrian sports’ governing bodies. 

Focus group participants highlighted areas where current performance evaluation and competition practices are not sufficient to safeguard horses’ welfare. The dressage group felt that, despite the rigorous training that judges receive, they do not protect horses from poor riding or poor welfare. This could be addressed by providing better training to allow judges to recognise and mark down behavioural signs that are indicative of conflict or underlying pain [46]. The dressage and showjumping groups recommended that stewards should observe warm-up areas to ensure horses are ridden appropriately outside of the competition arena. Providing/improving training for warm-up/collecting ring stewards could enable them to recognise and intervene in difficult situations where equine welfare is being compromised. Clear criteria for these interventions would be required to promote transparency and objectivity. Such interventions, which would be highly visible to both public and competitive stakeholders, could improve horse welfare and positively impact equestrian sports’ SLO. The endurance group felt that welfare issues exist at competitions that are not identified during the veterinary assessments. This suggests the need to improve the way in which welfare is monitored for the entire time a horse is at the competition venue, which could apply to all equestrian sporting disciplines. 

The immediate public visibility of different stages within each discipline varies, with show jumping and dressage competitions being held in highly visible enclosed arenas, and most endurance rides and parts of the cross-country section of eventing being relatively inaccessible. In the latter two cases, public scrutiny is focused on the horses as they finish or on any accidents or sudden collapses [47,48]. Many participants recognised that while improving competition practices could improve welfare during competitive events and could potentially improve the public perception of equestrian sport, competition represents only a very small part of a horse’s life. Although this is undoubtedly important in terms of equine welfare, the impact on the horse may be minimal in comparison with its general management and training. The basic needs of equine athletes are the same as for all horses, and the management styles under which many are kept often fail to satisfy these basic needs [4]. Factors associated with competitive sports, which include separation from the home environment and familiar equine companions [49], transportation [50] and housing in unfamiliar places, all contribute to compromised welfare [4]. 

It is unlikely that any changes made to regulations governing equestrian sport at competitions would have positive impacts on wider equine management issues in a horse’s home environment. Participants suggested that elite equine athletes are more likely to have a better standard of health, fitness and nutrition than those competing at a lower level and that they would score positively on any health-related welfare measure. Conversely, horses competing at a lower level were considered to be more likely to be ridden in ill-fitting/unsuitable tack and equipment. Additionally, they are more likely to be managed/ridden by less knowledgeable/experienced owners and riders but are often managed in a way which better provides for the ethological needs of the horse. Further exploration of the potential differences in welfare issues/standards for horses competing at the various levels is needed.

In addition to the specific points discussed above, several important themes emerged in relation to assessing the welfare of equine athletes. Participants’ understanding of welfare reflected many of the currently held scientific views on what is meant by the term. For example, many participants recognised the importance of meeting both emotional and physical needs [3], understood that both positive and negative affective states should be considered [8,11], and believed that assessment of whether a horse has a life worth living should be conducted over a prolonged period [8]. Assessing ‘welfare’ was perceived as problematic by participants, as the term was generally considered to be stigmatised due to its association with poor welfare. However, stakeholders were open to assessing related constructs, such as quality of life, as these were perceived to have more positive connotations. The strategic plan drawn up for the welfare of horses bred for racing includes reference to the need to provide all horses with a good quality of life [27]. Although “welfare” and “quality of life” are considered synonymous by many researchers, there is a temporal distinction, with quality of life usually being assessed over a longer period of time than welfare [28,29]. Replacing the term “welfare” with “quality of life” in the future development of assessment tools may cause them to be viewed more favourably, facilitating uptake by stakeholders and encouraging consideration over the lifetime of a horse [14]. 

Participants provided useful feedback on the feasibility of using formal/scientific tools to assess welfare. Although there was some support for such tools, it was unclear who should be carrying out these assessments and how they should be applied from a practical standpoint. Participants in this study discussed the need to consider individual differences when assessing behavioural signs of welfare; this was considered problematic due to the need to know the animal intimately enough to know what is “normal” for that horse yet be removed enough to be objective. Using a standardised method to assess a behavioural response can improve reliability but may not truly reflect what is normal for that individual horse, and this could affect uptake by end users [51]. Participants were unsure how well current welfare measures/tools account for individual differences in behaviour and responses to management and training that are apparent from an early age [52]. 

Health checks during competition are carried out in some equestrian sports, including endurance events [53], some levels of eventing and at the highest level of sporting events [54]. A team of people is involved in monitoring and maintaining the horse’s physical health, and the endurance group noted that the support crew in this sport may be more objective in this respect than the rider (who may be more focused on competitive success). Informal monitoring by the endurance crew was considered an important source of information about whether the horse is behaving ‘normally’ or whether there are signs of something being amiss. When deciding which stakeholders are most appropriate for use in a formal welfare assessment tool, researchers should consider all team members, not just the rider. However, in doing so, it is important to note that those stakeholders may also be influenced by the competitive environment and resultant complex ethical dilemmas [21,55].

Another key point made by participants in the eventing group was that the interpretation of certain behavioural responses needs to be contextualised. For example, signs of behavioural arousal at the start of a cross-country course may be considered as reflecting positive anticipation, or anxiety, depending on contextual factors. Although it is extremely difficult to develop a method for assessing a behavioural welfare measure that has good reliability and is flexible enough to account for context, the importance of how behaviour is interpreted should not be overlooked [56].

Our study shows that how the horse feels (its affective state) is not typically at the forefront of most participants’ thoughts in relation to competitive performance, although it is considered in other contexts. Participants suggested initiatives with the potential to avoid/reduce negative affective states at competitive events (for example, stewards observing the warm-up arenas to ensure that horses are ridden appropriately), but they spoke less frequently about providing horses with opportunities to experience positive affective states and, therefore, experience good welfare during competition. Avoiding/reducing negative affective states without providing opportunities to experience positive affective states can, at best, achieve a state of neutral welfare [6]. The promotion of positive welfare at competitive events could be one way to support welfare improvement, given that competitive success is a key motivating factor. For example, more deeply embedding measures of positive welfare into scoring could encourage competitors to implement change. The challenge is identifying what signs are indicators of positive welfare and communicating this to stakeholders. 

Participants were unsure as to how well current welfare measures/tools could assess affective states in training and competition. Some participants stated that signs of ‘happiness’ are currently unidentifiable. Accurately assessing equine emotional state is problematic because, while physiological measures (for example, heart rate) can provide an indicator of arousal levels, robust behavioural evidence of emotional valence is lacking [12]. Furthermore, when assessing ridden horse behaviour, stakeholders have been shown to draw different conclusions regarding underlying mental state [19]. Developing a valid and reliable means of assessing equine athletes’ emotional state would be a first step in understanding the true impacts management, training and competition practices have on horse welfare. Given the lack of validated behavioural measures of emotional state, the ability of existing over-arching welfare assessments to assess sports horses that may be physically, but not necessarily psychologically, healthy is unknown. 

Although there were representatives from different sporting disciplines, the nature of the workshop and focus group sessions will have resulted in a biased sample of participants. The sample was largely self-selected and, given the time commitment required and the prospect of discussion with animal welfare researchers, likely to already have an interest in equine welfare assessment within equestrian sport. Regardless, very few participants had used a training log or existing tool to monitor their horse’s welfare, which suggests that it is not common practice across the sector. Although current welfare assessment tools have been used to assess the welfare of equine athletes [57], their application to-date has been by researchers rather than by equine stakeholders. The results of this study suggest that existing welfare assessment tools are not currently considered feasible for use by stakeholders under real-life conditions. Consequently, researchers must involve all relevant stakeholders in the development of welfare assessment tools from an early stage and consider alternative approaches if they are to be utilized by end users. 

To facilitate discussion amongst stakeholders where there is conflict between the needs of the horse and the demands of competition, a scale of priorities for maintaining (and improving) equine welfare would be a useful point of reference. Welfare priorities within the general equine sector have been identified [58], and the current development of an ethical framework to help stakeholders make decisions about what should or should not be done in specific situations will be invaluable [55]. 

## 5. Conclusions

Issues relating to equine welfare at competitive events must be addressed to improve both the competitive experiences of the equine athletes and public perceptions of equestrian sport. However, as was noted repeatedly by participants, improvements in equine management and training are likely to have greater impacts on improving the welfare of equine athletes. Conflicting priorities of the different stakeholders, the demands of competition and the needs of the horse may result in compromised welfare. Current welfare assessment tools were not considered suitable for use in practice by stakeholders, so new approaches must be developed using a participatory process. 

## Figures and Tables

**Table 1:** Characteristics of participants within each of the focus groups (gender, competitive and welfare-related roles). FEI: Fédération Equestre Internationale.

Focus Groups	Participant Characteristics		
	Gender	Competitive Role	Animal Welfare Related Role (If Applicable)
Eventing	Male	Rider	Researcher
	Female	Rider	Researcher
	Male	FEI veterinarian	Researcher
	Female	Rider	Trainee behaviourist
	Female	Rider	Researcher
	Female	Owner	
	Female	Owner/rider	Researcher
	Female	Videographer	
Showjumping	Female	Rider	Researcher
	Female	Rider	Researcher
	Female	Rider	
	Female	Owner	Lecturer
	Female	Rider	Researcher
Dressage	Female	Coach	
	Female	Rider	Researcher
	Female	Spectator	Equine welfare charity
	Female	Researcher	
	Female	Veterinarian	Lecturer
	Female	Judge and coach	Lecturer
	Female	Rider	
	Female	Rider/working pupil	
	Female	Trainer, rider	
	Female	Rider	
Endurance	Female	Physiotherapist	
	Female	Rider	
	Female	Rider	
	Female	Rider	
	Female	Crew	
	Female	Rider	
Welfare	Female		Researcher
	Female		Researcher
	Female		Researcher
	Male		Animal welfare charity
	Female		Researcher
	Female		Equine welfare charity
	Female		Lecturer
	Male		Animal welfare charity
	Female		Researcher
	Female		Animal welfare charity
Mixed sports	Female	Competitive Western Rider	Researcher
	Female	Racing authority	Lecturer
	Male	Racing trainer	Racing authority
	Female	Competition coordinator	Riding instructor
	Female	Racing staff	Researcher
	Female		Researcher (racing)
	Female	Racing stud farm vet	Lecturer
	Female		Lecturer, Researcher
	Female	Polo	Researcher

## Data Availability

Anonymised data can be shared upon request with research organisations, subject to ethical approval. Please contact the authors for more information.

## Supplementary Materials

The following are available online at https://www.mdpi.com/article/10.3390/ani11113228/s1, Table S1: Workshop programme, Table S2: Focus groups plan.
