# Supplementary material for: How Happy Are Equine Athletes? Stakeholder Perceptions of Equine Welfare Issues Associated with Equestrian Sport
Source: Animals (Basel). 2021 Nov 12;11(11):3228. doi: 10.3390/ani11113228 (PMC8614509; doi:10.3390/ani11113228)
Supplement: Supplementary file 1 [file animals-11-03228-s001.zip › Table S1. Workshop Programme.pdf]

How Happy are Equine Athletes?
Assessing Equine Quality of Life in Equestrian Sporting Disciplines

PROGRAMME: 30th March 2021

09.00	Welcome	Dr Carol Hall (NEWC)
09.10	*Session 1: Setting the scene (Chair: Dr Carol Hall)*	
	Collaboration, consensus and communication	Dr David Marlin (President of NEWC)
	Social licence to operate	Roly Owers (World Horse Welfare)
	Ethical considerations in equestrian sport	Dr Madeleine Campbell (Royal Veterinary College)
	Q&A with the session speakers	
10.00	Break	
10.15	*Session 2: Equestrian sporting disciplines (Chair: Roly Owers)*	
	The happy equine athlete	Richard Davison<br>John McEwen
	Dressage	Charlotte Dicker
	Showjumping	Holly Smith
	Eventing	Sam York
	Endurance	Antonia Milner-Matthews
	Q&A with the session speakers	
12.00	Lunch	
12.45	*Session 3: Focus Group 1 (Introduction by Dr Tamzin Furtado)*	
	Focus Group 1	
13.45	Break	
14.00	*Session 4: Assessing equine behaviour (Chair: Dr David Marlin)*	
	Current approaches to assessing QoL in animals	Liane Preshaw (The Horse Trust)
	Behavioural evidence of the Fifth Domain	Dr Carol Hall (NEWC)
	Equine sleep patterns and equine wellbeing	Kym Griffin (Nottingham Trent University)
	Developing context specific QoL assessments	Dr Fiona Cooke (The Donkey Sanctuary)
	Q&A with session speakers	
15.15	Break	
15.30	*Session 5: Focus Group 2*	
	Focus Group 2	
16.40	Summary of Focus Group discussions	Dr Tamzin Furtado
16.55	Close of workshop	Dr Carol Hall
