# Supplementary material for: How Happy Are Equine Athletes? Stakeholder Perceptions of Equine Welfare Issues Associated with Equestrian Sport
Source: Animals (Basel). 2021 Nov 12;11(11):3228. doi: 10.3390/ani11113228 (PMC8614509; doi:10.3390/ani11113228)
Supplement: Supplementary file 1 [file animals-11-03228-s001.zip › Table S2. Focus groups plan.pdf]

Table S2. Focus group plan

Focus Group 1

Item and time activity should take total	Facilitator suggested wording	Facilitator instructions
Prepare		Open Jamboard, initiate recording
Intro<br>5 mins to get settled	• Thank you for taking part – I am [intro yourself]. So just to remind you, the idea behind these groups is to give you the chance to share your thoughts and experiences on the things discussed in the workshop so far.<br>• Obviously, everyone's opinion is their opinion and there's no right or wrong, so we aren't judging you and also, need to remember to be respectful of one another.<br>• We will also be recording the discussions, to provide information for future about the positives and negatives of equine quality of life in different equestrian disciplines.<br>• The group will be run under "Chatham House Rules" which means please keep things confidential within the group – this is a safe space to talk about our ideas about wellbeing, but that can be quite personal. Just as we all may not want our own thoughts broadcast, after the event, please don't share the names or stories of others who took part in the group<br>• If you want to talk, please raise your hand by using the "raise hand" button, which is on one of the "reactions" buttons there at the bottom of the screen. If you want to practice now, you can raise your hand – or give me a clap or smiley face if you want!<br>• Later we'll also use an online whiteboard, so you can answer the question by jotting down your thoughts while we're also talking. Don't worry if you can't use the online whiteboard – it is fine to just talk if you prefer – but it should help our discussion along, so if you're happy to give it a go then that would help us all. You can also use the "chat" function which is at the bottom of the screen if you want to add anything.	
Icebreaker (go round the table so everyone has a chance to speak)	This is the hardest bit of the whole discussion group, because I know you're all extremely experienced and have a long competition history, but unfortunately, we need to keep it short! – to start with, could you just tell us a little about yourself – just a sentence or two about you and your role in [discipline]	

5 mins		
Intro to Jamboard<br><br>5 mins	Thank you everyone.<br>So, for this task we are going to use an online whiteboard with virtual post-it notes." *Share screen with Jamboard* "Can everyone view my screen and see the Jamboard?<br><br>This is what we're going to be working on, and I'm going to share a link in the chat now that you need to click on to access the board." *share link in chat – you will be able to see when people are accessing the board* "We are going to be using the post-it notes, so when you access the board you need to navigate to the tool bar on the left-hand side, and go to the fourth icon down, which is a little square.<br><br>Then to add a post-it note you click on this icon and your post-it will pop up where you type your thoughts, and you can also choose the colour. They will all appear in the top left-hand corner of the screen, so you need to move them otherwise they pile up on top of each other." (Do all this on your share screen while saying this).<br><br>Please be aware that you can edit or delete other's notes, so be careful not to do this!"	Share screen with Jamboard – also add the link to the Jamboard in the chat
Q1<br><br>20 mins	Imagine you're a competition horse training in your discipline. What would make you a particularly **<u>happy</u>** athlete?<br><br>You can add your thoughts on the post-it notes on the board in front of you. Add as many as you like, and if you think things are interlinked, maybe put them near one another.<br><br>prompts:<br>• What would be the minimum acceptable versus "happy" athlete life?<br>• Do you as an equine athlete need different things in competition versus training versus management?<br>• Do you as an equine athlete have different needs to a leisure horse, or to horses in other disciplines?	Allow them to add post-its, move them around during the discussion if needed<br><br>Depending on what comes up, you could divide into:<br><br>Competition/management/training<br><br>Or:<br>Nutrition<br>Mental state<br>Behaviour<br>Physical wellbeing etc

Q2<br><br>10 mins	OK, now we are going to move on to the next question, and we don't need Jamboard for that so we can just see each other again.<br><br>The next point for discussion is:<br>What is being done within your discipline to promote happy equine athletes?<br>Prompts:<br>• Are you doing anything to promote happy equine athletes within your discipline?<br>• Is the national organisation (e.g. BD, BS etc) doing anything to promote happy equine athletes?<br>• What else could be done to make equine athletes happier?	Remove screenshare
Q3<br><br>14 mins	For this question, we are going to use Jamboard again [switch to new Jamboard] so back to the link in your browser if you're comfortable using it. The question this time is:<br><br>If you could change one thing to improve equine QOL for horses in your discipline what would it be?<br><br>Prompts:<br>• What might be publicly viewed as compromising the quality of life of equine athletes?<br>• Does this perception reflect the real issues from your perspective?<br>• What do you think are the main issues in terms of equine quality of life in your discipline?	Switch to new jamboard and share screen again (they use the same link)<br><br>This could be competition rules (e.g. having a maximum allowed speed in endurance, allowing a "buddy" horse in the ring for dressage) or at home
	Thank you for your time, it's been really great to meet you all. We'll be coming back together to talk about the issues in the second half of day, in the second focus group. I'll look forward to talking to you then!	Stop record, close Zoom meeting and allow zoom to save as recording.<br><br>Take photos of the Jamboards<br><br>Share with other facilitators, any notes about major themes/issues that emerged

Focus group 2

Item and time activity should take total	Facilitator suggested wording	Facilitator instructions
Re-introduction<br>1 min	Hi again everyone, thanks for coming back. This time, we are going to first discuss the things people said across different disciplines which promoted “happy athletes” within that discipline, and then talk about how you work out how happy your own horses are.	Initiate group and start recording
Q1<br><br>20 mins	First of all, if we go back to Jamboard, you can compare the different things that came up for each discipline. I’ll just give you a minute to look through that and compare with the things you said yourselves.<br><br>Looking at these, do any of the thoughts of people from other disciplines make you think differently about how things are done in your own?<br><br>Prompts:<br>• Is there anything from the other groups, that you think could work well for your discipline?<br>• Are there any issues highlighted in other disciplines which you feel are particularly concerning?	Share Jamboard screen, showing results of group 1
Q2<br><br>20 mins	When you have a horse in front of you, how do you decide if the horse is a happy athlete?<br><br>Prompts:<br>• do you use any formal monitoring with your own horses? Why/why not?<br>• Was there a time recently when you saw a really happy athlete (or were worried about an animal)? How did you know it was happy/unhappy?	Stop sharing screen
Q3<br><br>20 mins	What do you think about the use of formal assessments like the ones presented in the talk?<br><br>Prompts:<br>• Would you ever use these? When/how?	

	• What behaviours do you focus on in your assessments that tell you that a horse has a good/poor QoL within those contexts?<br>• Are there any discipline-specific behaviours or assessments should be included in an approach to assessing equine QoL in your discipline?<br>• Would an assessment be relevant to all disciplines, or is anything specific needed?	
Finish	Thank you so much for your time everyone – I have really enjoyed hearing your ideas, and think we all have some food for thought. We are now going to go back to the main meeting room to finish the meeting.	Stop recording and save the recording on your computer,<br>Write notes on immediate impressions, thoughts and themes
